# Increase in bile acids after sleeve gastrectomy improves metabolism by activating GPBAR1 to increase cAMP in mice with nonalcoholic fatty liver disease

**DOI:** 10.1002/iid3.1149

**Published:** 2024-07-19

**Authors:** Guoliang Li, Xin Xu, Lixin Chai, Qunhao Guo, Wei Wu

**Affiliations:** ^1^ Department of Gastrointestinal Hepatobiliary Surgery The Affiliated Hospital of Hangzhou Normal University Hangzhou City China

**Keywords:** bile acid, cAMP, GPBAR1, metabolism, nonalcoholic fatty liver disease

## Abstract

**Background:**

Bile acids (BAs) concentration can affect metabolic improvement caused by bariatric surgery and BA concentrations increase in patients after sleeve gastrectomy (SG). Here, how BAs after SG affect metabolism in nonalcoholic fatty liver disease (NAFLD) was studied.

**Methods:**

Mice were given high‐fat diet (HFD) to induce NAFLD and received SG surgery. Hepatic and fecal BA concentrations in mice were detected by liquid chromatography‐tandem mass spectrometry method. BA‐related genes were detected by quantitative real‐time polymerase chain reaction. G protein BA receptor 1 (GPBAR1) expression was identified using western blot analysis. NAFLD mice after SG received GPBAR1 inhibitor Triamterene. The weight of mice and mice liver was detected. Mouse liver tissue was observed by hematoxylin–eosin and Oil Red O staining. Triglyceride (TG), nonesterified fatty acid (NEFA), and cyclic adenosine monophosphate (cAMP) levels in mouse liver tissue were analyzed by metabolic assay and enzyme‐linked immune sorbent assay.

**Results:**

SG boosted increase in hepatic total/conjugated BAs and related genes and GPBAR1 expression, and attenuated increase in fecal total BAs/muricholic acid in HFD‐induced mice and increased fecal taurine‐BAs in HFD‐induced mice. Triamterene (72 mg/kg) reversed the inhibitory role of SG in HFD‐induced increase of body weight, lipid accumulation, inflammatory cell infiltration, and increase of hepatic weight and TG/NEFA content, and counteracted the positive role of SG in HFD‐induced increase of hepatic cAMP concentration in mice.

**Conclusions:**

BAs improve metabolism via activating GPBAR1 to increase cAMP in NAFLD mice after SG.

## Graphical abstract

(1) SG increased hepatic BAs in NAFLD mice. (2) SG improved metabolism in NAFLD mice. (3) GPBAR1 inhibitor aggravated metabolism in NAFLD mice after SG.

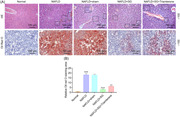

## 1 INTRODUCTION

Nonalcoholic fatty liver disease (NAFLD) is the most frequent chronic liver disease, which is characterized by hepatic parenchymal cell steatosis, intralobular inflammation as well as mild fibrosis without history of alcoholism, whereas other causes are excluded.^1^, ^2^ NAFLD is the liver manifestation of metabolic syndrome related to heredity, diabetes, dyslipidemia, insulin resistance, and obesity.^3^ In addition, NAFLD can develop from simple nonalcoholic fatty liver to nonalcoholic steatohepatitis, fibrosis, cirrhosis, hepatocellular carcinoma, and even death in severe cases.^1^, ^4^ Obesity is one of the independent risk factors for NAFLD and bariatric surgery is considered to be an effective method for losing weight in morbid obesity.^5^ Laparoscopic sleeve gastrectomy (LSG) is a surgical technique for weight loss, which can lead to marked weight loss with trifling complications, and there is no report of NAFLD aggravation after LSG.^6^ Our study aims to explore ways to improve the metabolism in NAFLD after LSG.

Previous studies have demonstrated bile acid (BA) concentrations in patients increase after sleeve gastrectomy (SG).^7^, ^8^ Primary BAs are synthesized from cholesterol in liver cells, and when conjugated with glycine or taurine, primary BAs are secreted through bile in the intestine where BAs promote the digestion and absorption of fat‐soluble vitamins and lipids.^9^ Primary BAs are deconjugated and dehydroxylated in the gut to form secondary BAs with most returning to the liver and a small part entering the systemic circulation or excreted through feces.^10^ Obesity‐related metabolic diseases, such as type 2 diabetes mellitus and NAFLD, have intimate association with dysregulation of BA homeostasis.^11^ At present, there is evidence proving that BA concentration may affect some metabolic improvement caused by weight loss surgery.^12^ In addition, the important role of BAs in NAFLD has been revealed in increasing studies.^13^, ^14^ However, how BAs after SG surgery affect metabolism in NAFLD remains unknown.

G protein BA receptor 1 (GPBAR1), also known as Takeda G protein‐coupled receptor 5 (TGR5), is a cell membrane receptor of BAs and BAs function in host immunity and metabolism through BA‐activated receptors, including GPBAR1.^15^, ^16^ In addition, BAs increase cyclic adenosine monophosphate (cAMP) content via specially activating BA receptor GPBAR1 and the BAs/GPBAR1/cAMP axis was reported to regulate energy expenditure.^17^ cAMP, a cyclic nucleotide, is an important intracellular second messenger affecting multiple cellular processes and cAMP signaling pathway may play an essential role for maintaining glucose metabolism.^18^, ^19^ Thus, we speculate BAs in NAFLD patients increase after SG to activate GPBAR1 and further induce cAMP production for alleviating NAFLD.

In this study, alterations in hepatic and fecal BA concentrations and hepatic GPBAR1 expression in NAFLD mice after SG operation were investigated. Then, how BAs and GPBAR1 affect metabolism in NAFLD after SG was studied in vivo.

## 2 MATERIALS AND METHODS

### 2.1 Animals

Male C57BL/6J mice (*n* = 90, 7 weeks old, ~22 g) were housed with a 12 h light/dark cycle at 22 ± 2°C and 40%–50% humidity. Standard laboratory food and water were provided for ad libitum consumption. All animal studies were approved by the Ethics Committee of Zhejiang Baiyue Biotech Co., Ltd for Experimental Animals Welfare (ZJBYLA‐IACUC‐20220701) and the guidelines of the China Council on Animal Care and Use were followed. Animals were kept for 1 week before experiments.

### 2.2 Animal model establishment, SG surgery, and administration

Mice were given a high‐fat diet (HFD, D12451, Research Diets; 45 kcal% saturated fat) to induce NAFLD or regular chow (5% fat, 53% carbohydrate, and 23% protein) for 12 weeks.^2^ For SG surgery, NAFLD mice were subjected to general inhalation anesthesia using 3% isoflurane (792632, Sigma‐Aldrich) and oxygen. In addition, 70%–80% of the lateral stomach was resected. A tubular gastric remnant in continuity with the pylorus and duodenum inferiorly and the esophagus superiorly was left. Sham surgery involved analogous stomach isolation followed by manual pressure application along a vertical line between the pylorus and esophageal sphincter with blunt forceps.^2^ A liquid diet (ENSURE, Abbott Laboratories) was provided to mice after the surgery for a 7‐day recovery period. In addition, mice were given gentamicin (HY‐A0276A, MedChemExpress) for 7 days after surgery. GPBAR1 inhibitor Triamterene (C_12_H_11_N_7_, 99.98%, HY‐B0575, MedChemExpress) was dissolved in dimethyl sulfoxide (D8371, Solarbio). After the 7‐day recovery period, mice were orally administered once with 72 mg/kg Triamterene.^20^

### 2.3 Grouping

In one trial, mice (*n* = 40) were randomly split into four groups (*n* = 10 in each group): the normal group, the NAFLD group, the NAFLD + sham group, and the NAFLD + SG group. Mice in the normal group received a regular chow, whereas those in other groups were given a HFD. Animals in the NAFLD + SG group were subjected to SG surgery and those in the NAFLD + sham group received sham surgery. After 6 weeks, mice were immediately subjected to general inhalation anesthesia using 3% isoflurane and oxygen, and were killed by cervical dislocation. Liver tissues were harvested and frozen in liquid nitrogen, and feces were collected as well. Both liver tissues and feces were stored at −80°C.

In another trial, mice (*n* = 50) were randomly split into five groups (*n* = 10 in each group): the normal group, the NAFLD group, the NAFLD + sham group, the NAFLD + SG group, and the NAFLD + SG + Triamterene group. Mice in the normal group, the NAFLD group, the NAFLD + sham group, and the NAFLD + SG group were treated as described above and those in the NAFLD + SG + Triamterene group were administered with 72 mg/kg Triamterene after NAFLD modeling and SG surgery. The weight of mice in each group was documented before operation and weekly after operation for 6 weeks. Subsequently, mice were anesthetized and killed as described above. The livers of mice were weighed and collected liver tissues were frozen in liquid nitrogen and stored at −80°C.

### 2.4 Detection of hepatic and fecal BAs

BA standards, including taurolithocholic acid (TLCA, T7515), taurodeoxycholic acid (TDCA, T0557), ursodesoxycholic acid (UDCA, 1707806), and tauro‐β muricholic acid (T‐βMCA, 700244P) were obtained from Sigma‐Aldrich. Lithocholic acid (LCA, MB5383), cholic acid (CA, MB5177), taurochenodeoxycholic acid (TCDCA, MB7040), deoxycholic acid (DCA, MB5278), and chenodeoxycholic acid (CDCA, MB5978) were provided by Meilun. Tauroursodeoxycholic acid (TUDCA, 496672) was purchased from J&K Scientific. Β muricholic acid (βMCA, B74541) and taurocholic acid (TCA, B26949) were provided by Yuanye. Deuterated CA‐2,2,4,4‐d_4_ was purchased from C/D/N Isotopes.

For BA evaluation, 1 mL of ddH_2_O with CA‐d_4_ (200 ng) and DHCA (40 ng) was used to homogenize liver tissues and fecal samples, which were then centrifuged for 10 min at 14,000*g*, 4°C. In addition, the supernatant was loaded onto a preactivated Hypersep C18 solid phase extraction column (Thermo Fisher). Subsequently, the column was added with 1 mL of ddH_2_O for the removal of impurities, and the analytes were eluted using 1 mL of methanol (721964, Sigma‐Aldrich). The collected methanolic solution was evaporated to dryness at 37°C under a stream of nitrogen gas. The dried extract was reconstituted with 100 μL mobile phase from which an Agilent 1290 system (Agilent Technologies) coupled with an AB Sciex 6500 QTRAP ® mass spectrometer equipped with an electrospray ionization source was injected with 1 μL of the sample. In addition, the liquid chromatography‐tandem mass spectrometry method was as described in a previous study.^21^

### 2.5 Western blot analysis

Liver tissues were lysed in a radioimmunoprecipitation assay lysis buffer (P0013C, Beyotime). Protein concentrations were measured using a BCA Protein Assay Kit (7780S, CST). Proteins were separated by sodium dodecyl sulfate polyacrylamide gel electrophoresis (P0012A, Beyotime) and transferred to polyvinylidene difluoride membranes (88518, Thermo Fisher). The membranes were blocked for 2 h by 5% skim milk at room temperature and then incubated overnight at 4°C with the primary antibody against GPBAR1 (ab72608, 33 kDa, 1:1000, Abcam) or the inner control glyceraldehyde‐3‐phosphate dehydrogenase (GAPDH) (ab9485, 36 kDa, 1:2500, Abcam). After washing with Tris‐buffered saline with 0.1% Tween‐20 (T1081, Solarbio), membranes were incubated for 1 h with Goat Anti‐Rabbit IgG (AP132, 1:3000, Sigma‐Aldrich) at room temperature. The chemiluminescent signals were detected with Chemiluminescent Substrate (34577, Thermo Fisher) using the Odyssey® M Imaging System (LI‐COR Biosciences). In addition, the results were analyzed using ImageJ software (National Institutes of Health).

### 2.6 Quantitative real‐time polymerase chain reaction (qRT‐PCR)

The RNA prep pure tissue kit (12033674001, Roche) was used to extracted total RNA from the liver tissues. qRT‐PCR reactions were performed BeyoFast™ SYBR Green One‐Step qRT‐PCR Kit (D7268S, Beyotime) under a PCR system (7300 Real‐Time PCR System, Applied Biosystems). GAPDH was taken as the normalization control. These gene expressions were counted by the 2^−ΔΔCt^ method. The primers were as follow: cholesterol 7α‐hydroxylase (CYP7A1) (forward: 5′‐CAAGCAAACACCATTCCAGCGAC‐3′, reverse: 5′‐ATAGGATTGCCTTCCAAGCTGAC‐3′); cholesterol 27‐hydroxylase (CYP27A1) (forward: 5′‐GTGCTGCCTTTCTGGAAGCGAT‐3′, reverse: 5′‐TAGCCAGACACCTGGATGCCAT‐3′); farnesoid X receptor (FXR) (forward: 5′‐TGGGTACCAGGGAGAGACTG‐3′, reverse: (5′‐GTGAGCGCGTTGTAGTGGTA‐3′); LXRα (forward: 5′‐AAGCCCTGCATGCCTACGT‐3′, reverse: 5′‐TGCAGACGCAGTGCAAACA‐3′); GAPDH, (forward: 5′‐TGACCTCAACTACATGGTCTACA‐3′, reverse: 5′‐CTTCCCATTCTCGGCCTTG‐3′.

### 2.7 Hematoxylin–eosin (HE) staining

Liver tissues were fixed in 10% neutral formalin solution (G2161, Solarbio), followed by decalcification using 5% nitric acid (438073, Sigma‐Aldrich) and dehydration in 70%–95% ethanol (E111991, Aladdin). The samples were then clarified with xylene (X112054, Aladdin) and embedded in paraffin, and 5 μm‐thick sections were prepared. Following deparaffin and treatment with 95%–70% ethanol, 1 mL of hematoxylin (H, H8070, Solarbio) was used to stain the sections for 8 min. Subsequently, the sections were differentiated in 5% acetic acid solution (A291611, Aladdin) and rinsed in running water, followed by treatment with 0.5% Ammonium Hydroxide (A299569, Aladdin) and another rinse. And 0.5% eosin (E, E292725, Aladdin) was used to stain the sections for 3 min. The pathological changes were observed under a light microscope (WMS‐1033, WUMO) at ×100 magnification.

### 2.8 Oil Red O staining

Histological visualization of lipid deposition in liver tissues was performed using an Oil Red O Staining Kit (C0157S, Beyotime). In brief, the frozen liver tissues were fixed in precooled 4% paraformaldehyde (C104190, Aladdin) for 20 min at room temperature, washed three times in cold water, and stored at −20°C. The samples were then sectioned in 5 μm and the sections were fixed in 4% paraformaldehyde for 30 min, washed, and stored at −20°C. To prepare Oil Red O working solution, the Oil Red O solution was diluted in Oil Red O diluent in a 3:2 ratio. The sections were warmed for 10 min, covered for 20 s with stain‐cleaning solution, and stained for 20 min by Oil Red O working solution at room temperature. Stain‐cleaning solution was added after the removal of Oil Red O working solution, followed by standing for 30 s. Stain‐cleaning solution was removed, followed by washing in distilled water for 20 s. The sections were counterstained with H and observed under a light microscope at ×100 magnification.

### 2.9 Metabolic assay and enzyme‐linked immune sorbent assay (ELISA)

The contents of triglyceride (TG) and nonesterified fatty acid (NEFA) in mouse liver were detected using kits (290‐63701, 294‐63601, Wako). For TG assessment, samples were incubated with the color reagent. In addition, the absorbance was measured using a microplate reader (1215D29, Thomas Scientific) at 600 nm (dominant wavelength) and 700 nm (subwavelength). For NEFA detection, each well was incubated with 80 μL Color Reagent A for 10 min at 37°C after 4 μL sample or standard was added to appropriate wells, followed by incubation for 10 min with 160 μL Color Reagent B at 37°C. After cooling the reaction solution to room temperature, the absorbance was measured using a microplate reader at 550 nm. cAMP level in liver tissue was evaluated using an ELISA kit (ab65355, Abcam). Prepared samples and standards were added to tubes, followed by adding neutralizing buffer. Tubes were incubated with acetylating reagent mix for 10 min at room temperature, and assay buffer was added. After transfer to protein‐G 96‐well plate, wells were incubated with cAMP antibody for 1 h and cAMP‐HRP for 1 h at room temperature. After washing with assay buffer, wells were incubated with HRP developer for 1 h. The reaction was stopped with HCl, and the absorbance at 450 nm was measured using a microplate reader.

### 2.10 Statistical analysis

Statistical analysis was performed using Graphpad Prism 8.0 software (Graphpad Software). Data were reported as mean ± SD. Comparisons among multiple groups were conducted using one‐way analysis of variance. Differences were considered as significant when *p* < .05.

## 3 RESULTS

### 3.1 SG promoted increase in hepatic BAs and its related genes and GPBAR1 expression, and mitigated increase in fecal BAs in HFD‐induced mice

**Figure 1:**
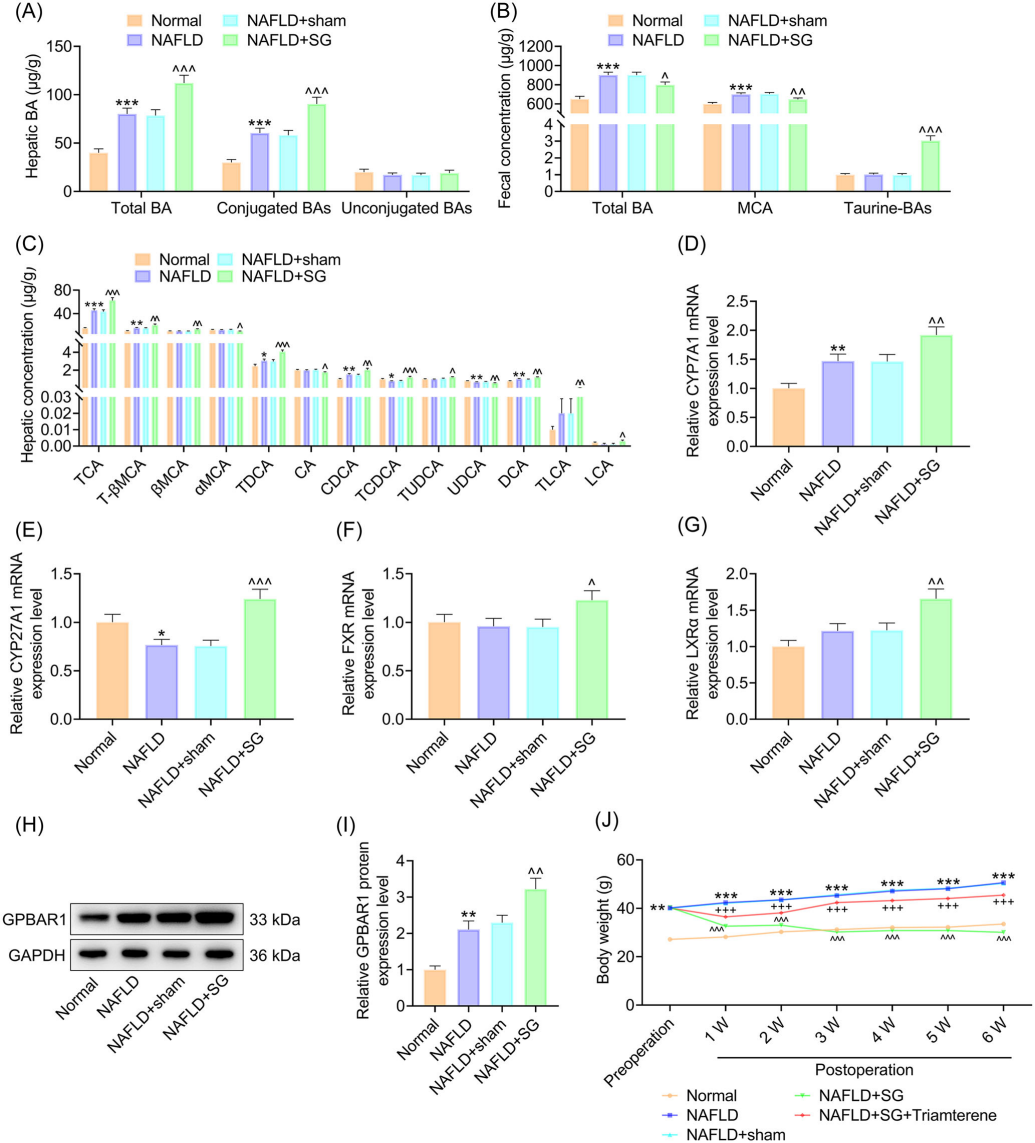
SG promoted increase of hepatic BAs and GPBAR1 level, and mitigated increase of fecal BAs, and Triamterene reversed inhibitory role of SG in increase of weight in HFD‐induced mice. (A–E) Mice were given a regular chow (normal) or HFD (NAFLD) for 12 weeks. NAFLD mice received SG surgery or sham surgery or not. (A) Concentrations of hepatic total BAs, conjugated BAs, and unconjugated BAs in mice were detected using the LC‐MS/MS method. (B) Concentrations of fecal total BAs, MCA, and taurine‐BAs in mice were analyzed using the LC‐MS/MS method. (C) Concentrations of hepatic BAs in mice were detected using the LC‐MS/MS method. (D–G) The expression of BA‐related genes in liver was detected by using qRT‐PCR. (H, I) GPBAR1 expression in mouse liver tissue was identified by western blot analysis and GAPDH was used as an internal standard. (J) Mice were given a regular chow (normal) or HFD (NAFLD) for 12 weeks. NAFLD mice received SG surgery or sham surgery, or not. In addition, NAFLD mice after SG operation were orally administered once with Triamterene (72 mg/kg) or not. The weight of mice in each group was detected before and after SG surgery. *p < .05, **p < .01, ***p < .001 versus Normal; ^^^ *p* < .05, ^^^^ *p* < .01, ^^^^^ *p* < .001 versus NAFLD+sham; ^+++^ *p* < .001 versus NAFLD + SG. BA, bile acid; CA, cholic acid; CDCA, chenodeoxycholic acid; GAPDH, glyceraldehyde‐3‐phosphate dehydrogenase; GPBAR1, G protein BA receptor 1; HFD, high‐fat diet; LCA, lithocholic acid; LC‐MS/MS, liquid chromatography‐tandem mass spectrometry; MCA, muricholic acid; NAFLD, nonalcoholic fatty liver disease; qRT‐PCR, quantitative real‐time polymerase chain reaction; SG, sleeve gastrectomy; T‐βMCA, tauro‐β muricholic acid; TCA, taurocholic acid; TCDCA, taurochenodeoxycholic acid; TDCA, taurodeoxycholic acid; TLCA, taurolithocholic acid; TUDCA, tauroursodeoxycholic acid; UDCA, ursodesoxycholic acid.

To explore how BAs affect NAFLD after SG, C57BL/6J mice received a regular chow (normal) or HFD (NAFLD) for 12 weeks and NAFLD mice were subjected to SG surgery or sham surgery as the control. Targeted metabolomic analysis of BAs was conducted in the liver and fecal samples from mice, and we found compared with the normal mice, NAFLD mice presented obviously upregulated total and conjugated hepatic BA levels (Figure 1A, *p* < .001). Increase in the total and conjugated hepatic BA levels in HFD‐fed mice was further boosted after SG surgery (Figure 1A, *p* < .001). Mice in the NAFLD group displayed increased fecal concentrations of total BA and MCA (Figure 1B, *p* < .001), while SG surgery attenuated increase in the fecal total BA and MCA concentrations in HFD‐fed mice (Figure 1B, *p* < .05). Additionally, the fecal concentrations of taurine‐BAs in NAFLD mice were increased after SG surgery (Figure 1B, *p* < .001). Statistical analyses in Figure 1C further exhibited the hepatic concentrations of TCA, T‐βMCA, TDCA, CDCA and DCA were increased while the hepatic concentrations of TCDCA and UDCA were decreased in NAFLD mice (Figure 1C, *p* < .05). SG upregulated hepatic TCA, T‐βMCA, βMCA, TDCA, CDCA, TCDCA, TUDCA, DCA, TLCA, and LCA contents, and downregulated hepatic αMCA, CA, and UDCA in HFD‐fed mice (Figure 1C, *p* < .05). Then, to investigate the regulatory mechanisms of BAs induced by SG treatment, mRNA expression of enzymes involved in BA synthesis (including CYP7A1 and CYP27A1) and nuclear receptors (including FXR and LXRα) were measured in the study. The expression of CYP7A1 and LXRα was increased, and the expression of CYP27A1 was decreased in the NAFLD mice. However, SG surgery increased the expression of CYP7A1, CYP27A1, FXR, and LXRα (Figure 1D–G, *p* < .05). Furthermore, western blot analysis results showed hepatic GPBAR1 expression was enhanced in HFD‐fed mice compared with the normal mice (Figure 1H–I, *p* < .01) and GPBAR1 level in NAFLD mice was upregulated after SG (Figure 1I, *p* < .01). Taken together, SG promoted increase in hepatic BAs and its related genes and GPBAR1 expression and mitigated increase in fecal BAs in NAFLD mice.

### 3.2 Triamterene reversed inhibitory role of SG in HFD‐induced increase of mouse weight

To investigate how GPBAR1 affects NAFLD after SG, GPBAR1 inhibitor Triamterene (72 mg/kg) was given to NAFLD mice after SG surgery. The mice had obviously higher body weight after 12‐week HFD feeding (Figure 1J, *p* < .01). SG operation decreased the weight of HFD‐fed mice (Figure 1J, *p* < .001), which was reversed by Triamterene treatment (Figure 1J, *p* < .001). Therefore, Triamterene reversed inhibitory role of SG in HFD‐induced increase of mouse weight.

### 3.3 Triamterene reversed inhibitory role of SG in HFD‐induced NAFLD symptoms in mice

**Figure 2:**
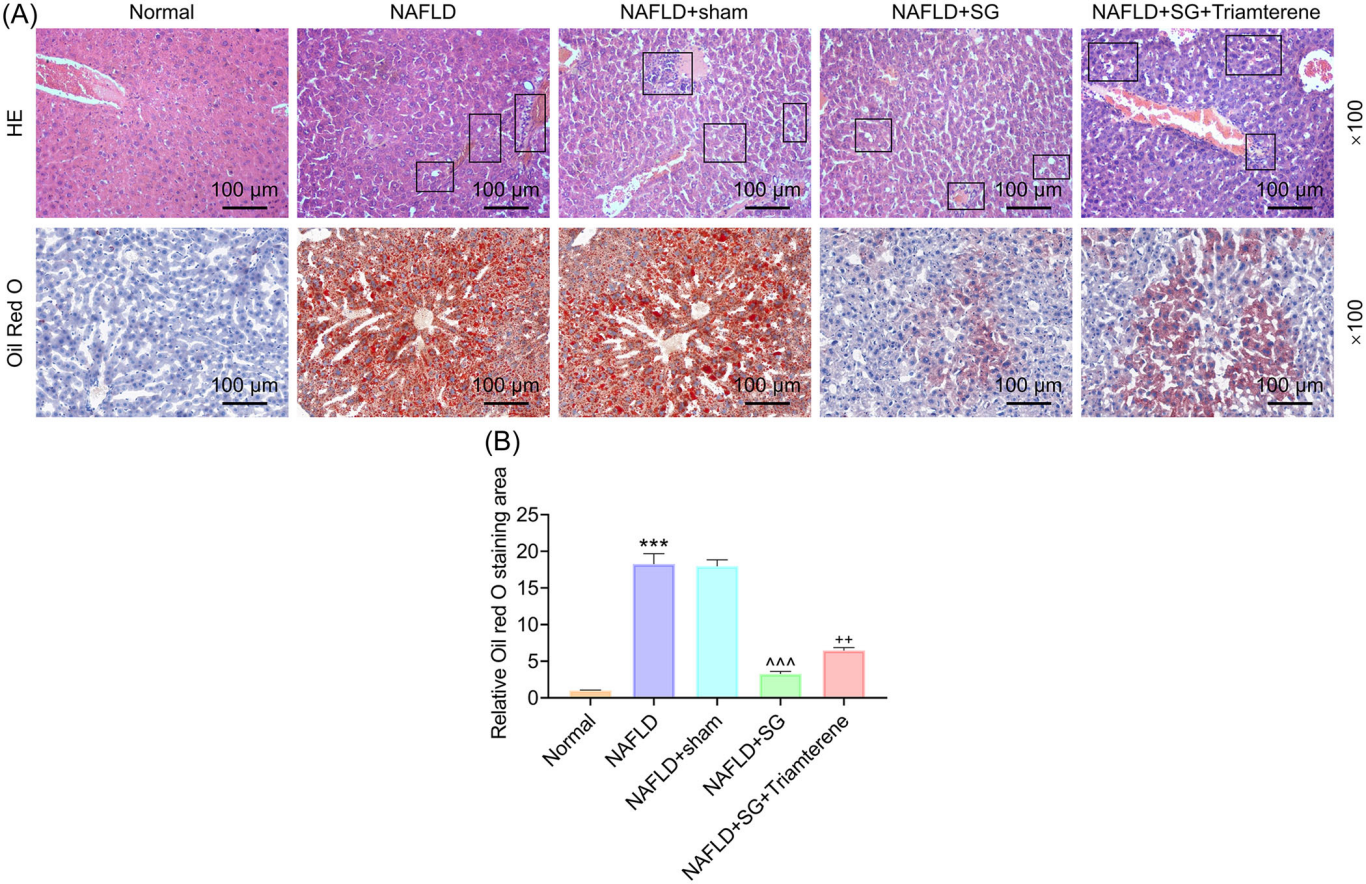
Triamterene reversed inhibitory role of SG in HFD‐induced NAFLD symptoms in mice. (A) Mice were given a regular chow (normal) or HFD (NAFLD) for 12 weeks. NAFLD mice received SG surgery or sham surgery or not. And NAFLD mice after SG operation were orally administered once with Triamterene (72 mg/kg) or not. Histopathological characteristics of mouse liver tissue were visualized using HE and Oil Red O staining. Scale bar = 100 μm, magnification, ×100. (B) The quantification results of oil red O staining. ***p < 0.001 versus Normal; ^^^^^ *p* < .001 versus NAFLD + sham; ^++^ *p* < .01 versus NAFLD + SG. HE, hematoxylin‐eosin; HFD, high‐fat diet; NAFLD, nonalcoholic fatty liver disease; SG, sleeve gastrectomy.

According to HE and Oil Red O staining, lipid accumulation and inflammatory cell infiltration were induced in HFD‐fed mice compared with the normal mice (Figure 2A,B). For HE assay, there was no obvious pathological phenomenon in the Normal group, and fat accumulation and inflammatory infiltration was observed in group NAFLD, similar in the NAFLD + sham group. In addition, fat accumulation and inflammatory cell infiltration were significantly reduced after SG surgery (NAFLD + SG group); however, this was revised by Triamterene (NAFLD + SG + Triamterene group). This was also confirmed by oil red O staining and its quantification results (Figure 2B). Collectively, SG surgery markedly attenuated lipid accumulation and infiltration of inflammatory cells in NAFLD mice, and Triamterene counteracted the inhibitory role of SG in HFD‐induced NAFLD symptoms in mice (Figure 2A,B).

### 3.4 Triamterene reversed inhibitory role of SG in HFD‐induced increase of hepatic weight, TG, and NEFA, and its positive role in HFD‐induced increase of hepatic cAMP in mice

**Figure 3:**
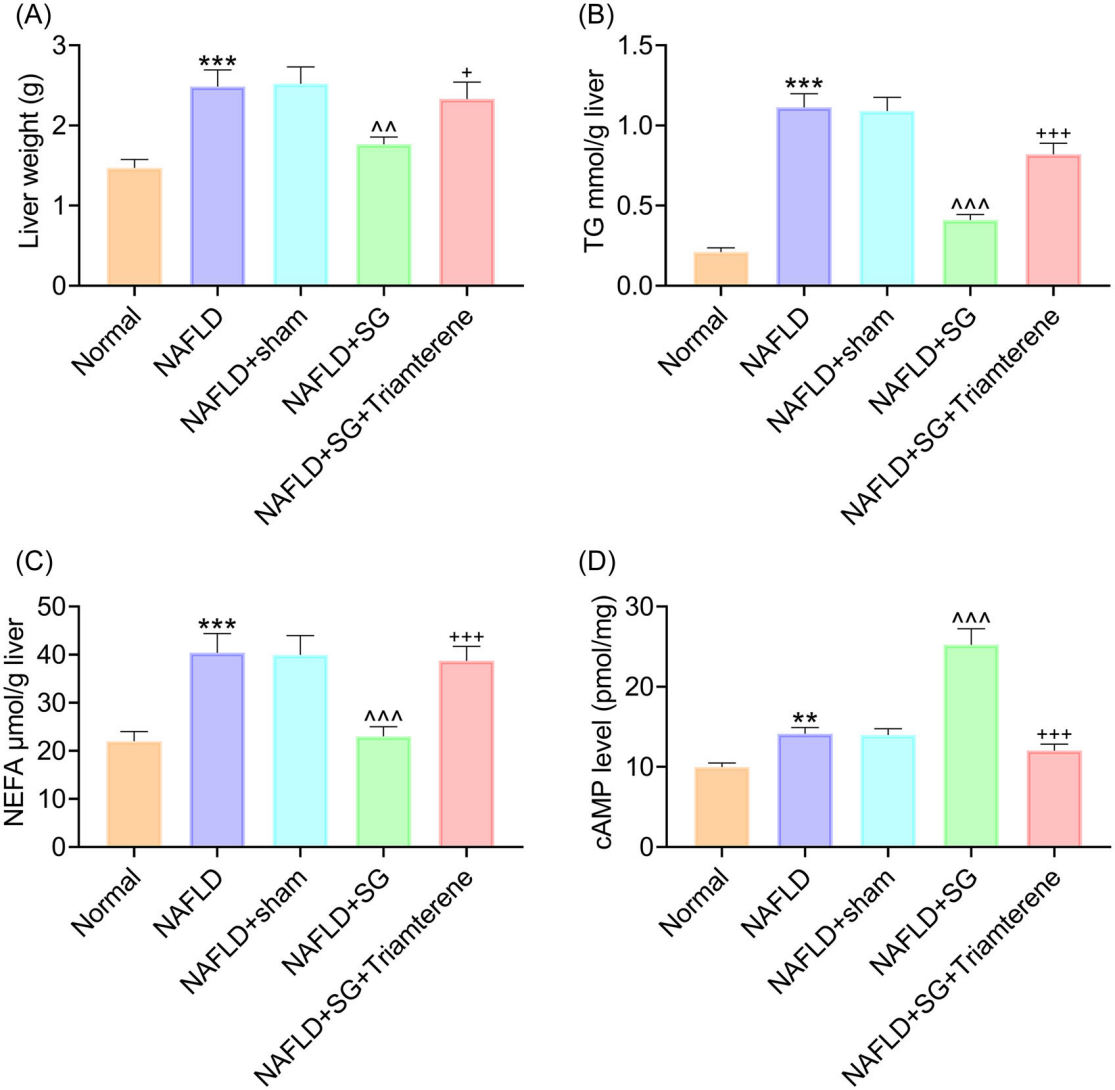
Triamterene reversed inhibitory role of SG in HFD‐induced increase of hepatic weight, TG, and NEFA, and its positive role in HFD‐induced increase of hepatic cAMP in mice. (A–D) Mice were given a regular chow (normal) or HFD (NAFLD) for 12 weeks. NAFLD mice received SG surgery or sham surgery or not. In addition, NAFLD mice after SG operation were orally administered once with Triamterene (72 mg/kg) or not. (A) Liver weight of mice was detected. (B, C) The concentrations of TG and NEFA in mouse liver tissue were measured by metabolic assay. (D) cAMP level in mouse liver tissue was detected by ELISA. **p < .01, ***p < .001 versus Normal; ^^^^ *p* < .01, ^^^^^ *p* < .001 versus NAFLD + sham; ^+^ *p* < .05, ^+++^ *p* < .001 versus NAFLD + SG. cAMP, cyclic adenosine monophosphate; ELISA, enzyme‐linked immune sorbent assay; HFD, high‐fat diet; NAFLD, nonalcoholic fatty liver disease; NEFA, nonesterified fatty acid; TG, triglyceride; SG, sleeve gastrectomy.

Liver weight of NAFLD mice was higher than that of the normal mice (Figure 3A, *p* < .001) and SG operation reduced liver weight of HFD‐fed mice (Figure 3A, *p* < .01). However, Triamterene treatment made liver weight of NAFLD mice after SG surgery higher (Figure 3A, *p* < .05). Compared with the normal mice, NAFLD mice showed more TG and NEFA in hepatic tissue (Figure 3B,C, *p* < .001). Increase in hepatic TG and NEFA contents in NAFLD mice was mitigated by SG operation (Figure 3B,C, *p* < .001), which was reversed by Triamterene (Figure 3B,C, *p* < .001). Furthermore, cAMP level in liver tissue from mice was upregulated after 12‐week HFD feeding (Figure 3D, *p* < .01), which was further promoted by SG surgery (Figure 3D, *p* < .001). In addition, Triamterene downregulated cAMP level in liver tissue from NAFLD mice after SG (Figure 3D, *p* < .001). Consequently, Triamterene reversed inhibitory role of SG in increase of hepatic weight, TG, and NEFA, and its positive role in increase of hepatic cAMP in NAFLD mice.

## 4 DISCUSSION

NAFLD is a liver disease associated with metabolic syndrome and obesity, characterized by hepatic cell dysfunction, inflammation, and hepatic fat accumulation.^22^ Although there are many research about NAFLD, a lack of effective therapeutic strategies and the complex pathogenesis contribute to a high incidence of NAFLD.^23^ Bariatric surgery is the most effective method for weight loss in morbid obesity, and there is no evidence to support NAFLD deterioration after LSG.^2^ Thus, ways to improve metabolism in NAFLD after SG were studied. In addition, mice were fed with HFD for 12 weeks to induce NAFLD and were subjected to SG surgery in the present study. Here we found SG promoted increase in hepatic BAs in HFD‐induced mice.

BAs play a crucial part in regulating energy, glucose, and lipid metabolism, and metabolic diseases related to obesity, including NAFLD, are associated with dysregulated BA homeostasis.^11^ For example, Ge et al.^24^ reported diets with higher BA levels improved growth performance and lipid metabolism in broilers. Maintaining metabolic homeostasis of BAs has protective effect on HFD‐induced NAFLD in mice,^25^ promote the excretion of BA, and improve the lipid metabolism disorder induced by high fat in NAFLD mice.^26^ Many studies have reported that metabolic surgery and its metabolic improvement are related to elevated BAs in the circulation. A study on rodents reported that increased serum total BA after vertical SG.^27^ Moreover, patients after SG operation displayed higher BA concentrations in previous studies.^7^, ^8^ Chen et al.^28^ reported many BA species were increased as early as 3 days postoperation with SG, which are sustained at 3 months postoperation.

Nevertheless, the regulatory role of changes in BAs after SG surgery in metabolism in NAFLD remains unclear. NAFLD is characterized by TG deposition in liver and NEFA accumulation in hepatocytes.^29^, ^30^ Further, we found that SG inhibited increase of body weight, lipid accumulation, inflammatory cell infiltration, and increase of hepatic weight and TG and NEFA levels in HFD‐induced mice. CYP7A1, CYP27A1, FXR, and LXRα were plays an important role in the liver BAs synthesis.^31^, ^32^ Several studies have shown that the expression of CYP7A1 and LXRα was increased, CYP27A1 and FXR was decreased in the NAFLD model,^14^, ^33^ and our result indicated that CYP7A1, CYP27A1, FXR, and LXRα expression were increased after SG treatment. These results suggested increase in BAs after SG improved metabolism in NAFLD. Previous study showed that the GPBAR1‐dependent anti‐inflammatory effects during cholestasis may occur downstream of BA‐mediated hepatocyte inflammation.^34^ In addition, we then indicated that GPBAR1 expression in liver tissue was increased after SG.

GPBAR1 is one of the major receptors for BAs.^35^ Policosanol alleviates hepatic lipid accumulation by regulating BA metabolism through the activation of GPBAR1.^36^ FXR induces GPBAR1 cross‐talk to regulate BA synthesis and improve hepatic lipid and glucose metabolism in obese mice induced by HFD.^37^ Previous study showed that after vertical sleeve gastrectomy surgery, there was no change in the composition of BAs in TGR5‐deficient mice.^38^ Further, we found in this study is that GPBAR1 inhibitor Triamterene counteracted the inhibitory effect of SG on increase of body weight, lipid accumulation, inflammatory cell infiltration, as well as increase of hepatic weight, and TG and NEFA contents in HFD‐fed mice. Thus, GPBAR1 signaling expression is necessary to confer a positive therapeutic outcome of the procedure. Furthermore, BAs increase cAMP content through the activation of its receptor GPBAR1 and the BAs/GPBAR1/cAMP signaling pathway was demonstrated to mediate energy consumption, and finally activates protein kinase A.^17^, ^39^ Reduced noradrenergic cAMP is related to dysregulated cell metabolism.^40^ And our results presented GPBAR1 inhibitor Triamterene reversed the positive effect of SG on increase of hepatic cAMP content in HFD‐induced mice. Besides, GPBAR1 inhibits the transcription of NF‐kB‐mediated pro‐inflammatory cytokines (interleukin [IL]‐1α, IL‐1β, tumor necrosis factor‐α, and so on), and maintains the expression of anti‐inflammatory cytokines (IL‐10) through cAMP pathway.^41^ Therefore, the above findings indicated increased BAs after SG improved metabolism in NAFLD through the GPBAR1/cAMP axis.

However, there are some limitations in this study, we should design Normal + SG group to explore what is the effect of SG surgery on BA levels and GPBAR1 receptors in normal mice.

To sum up, increased BAs after SG improved metabolism in NAFLD by promoting cAMP expression through the activation of GPBAR1, which provides theoretical reference for NAFLD treatment with BAs after SG surgery. In addition, this study investigated the effect of BA–BA receptor pathway on metabolism in NAFLD after SG for the first time. However, more downstream targets of BAs involved in the mechanism of BAs after SG in NAFLD need further exploration.

## AUTHOR CONTRIBUTIONS

Guoliang Li, Xin Xu, Lixin Chai, Qunhao Guo and Wei Wu performed the research. Guoliang Li designed the research study. Xin Xu, Lixin Chai, Qunhao Guo and Wei Wu analyzed the data. Guoliang Li, Xin Xu, Lixin Chai, Qunhao Guo and Wei Wu wrote the paper. Guoliang Li, Xin Xu, Lixin Chai, Qunhao Guo and Wei Wu have read and approved the final manuscript.

## CONFLICT OF INTEREST STATEMENT

The authors declare no conflict of interest.

## Data Availability

The analyzed data sets generated during the study are available from the corresponding author on reasonable request.
